# Harnessing systems biology approach for characterization of carotenoid biosynthesis pathways in microalgae

**DOI:** 10.1016/j.bbrep.2024.101759

**Published:** 2024-06-21

**Authors:** Bahman Panahi, Nahid Hosseinzadeh Gharajeh, Hossein Mohammadzadeh Jalaly, Mohammad Amin Hejazi

**Affiliations:** a Department of Genomics, Branch for Northwest & West Region, Agricultural Biotechnology Research Institute of Iran (ABRII), Agricultural Research, Education and Extension Organization (AREEO), Tabriz, Iran; b Department of Plant Breeding and Biotechnology, Faculty of Agriculture, University of Tabriz, Tabriz, Iran; c Department of Food Biotechnology, Branch for Northwest & West Region, Agricultural Biotechnology Research Institute of Iran (ABRII), Agricultural Research, Education and Extension Organization (AREEO), Tabriz, Iran

**Keywords:** Microalgae, Carotenoid, System biology, Pathway, Networks

## Abstract

Systems biology is an interdisciplinary field that aims to understand complex biological processes at the system level. The data, driven by high-throughput omics technologies, can be used to study the underpinning mechanisms of metabolite production under different conditions to harness this knowledge for the construction of regulatory networks, protein networks, metabolic models, and engineering of strains with enhanced target metabolite production in microalgae. In the current study, we comprehensively reviewed the recent progress in the application of these technologies for the characterization of carotenoid biosynthesis pathways in microalgae. Moreover, harnessing integrated approaches such as network analysis, meta-analysis, and machine learning models for deciphering the complexity of carotenoid biosynthesis pathways were comprehensively discussed.

## Highlights

• The data, driven by high-throughput omics technologies, can be used to study the underpinning mechanisms of metabolite production under different conditions.
• We comprehensively reviewed the recent progress in the application of these technologies for the characterization of carotenoid biosynthesis pathways in microalgae.
• Harnessing integrated approaches such as network analysis, meta-analysis, and machine learning models for deciphering the complexity of carotenoid biosynthesis pathways were discussed.

## 1 Introduction

Carotenoids, including β-carotene, astaxanthin, lutein, and fucoxanthin, are highly valued for their diverse range of applications in the food, supplement, pharmaceutical, and feed industries due to their coloring and nutraceutical properties [1]. These bioactive compounds possess strong antioxidant activities, which contribute to their health benefits, including the prevention of chronic diseases such as cardiovascular, neurological, metabolic, and cancer diseases [2,3]. By neutralizing reactive oxygen species (ROS) and nitrogen species, carotenoids act as antioxidants and protect against oxidative stress, thus reducing the risk of degenerative disorders. Research has shown that carotenoids have various biological activities, such as anti-tumor, anti-diabetic, anti-aging, and anti-inflammatory properties, making them promising candidates for functional health foods and nutraceuticals that promote human health. Despite their numerous advantages, efforts are currently being made to improve the bioavailability of carotenoids from underutilized foods, with the aim of maximizing their absorption and bioactivity for improved health outcomes [4].

Systems biology is the interdisciplinary field of computational modeling, molecular biology, and biochemistry that aims at the system-level understanding of biological processes and the identification of genes and functions of their products through large-scale analysis of genotype-phenotype relationships [5,6]. High-throughput omics technologies such as genomics, transcriptomics, proteomics, metabolomics, and ionomics are of central importance to the system biology field [7]. Table 1 shows a list of different –omics studies on carotenoid synthesis in microalgae. The data driven from these technologies can be used to study the underpinning mechanisms of metabolite production under different conditions to harness this knowledge for the construction of gene networks, protein networks, metabolic models, and engineering of strains with enhanced target metabolite production in microalgae.

**Table 1:** The list of references for genomic, transcriptomics, proteomics and integrative studies on carotenoid synthesis in microalgae.

Approach	Subject of study	Reference
Genomics	Genome-based identification and comparative analysis of enzymes for carotenoid biosynthesis in microalgae	(Narang et al., 2022)
	Genomic analysis of mutants affecting xanthophyll biosynthesis and regulation of photosynthetic light harvesting in Chlamydomonas reinhardtii	(Anwaruzzaman et al., 2004)
	Draft nuclear genome sequence of the halophilic and beta-carotene-accumulating green alga Dunaliella salina strain CCAP19/18	(Polle et al., 2017)
	Genome Sequence of the Oleaginous Green Alga, Chlorella vulgaris UTEX 395	(Guarnieri et al., 2018)
	Genome sequencing, assembly, and annotation of the self-flocculating microalga Scenedesmus obliquus AS-6-11	(Chen et al., 2020)
Transcriptomics	De novo transcriptome analysis of an aerial microalga Trentepohlia jolithus: pathway description and gene discovery for carbon fixation and carotenoid biosynthesis	(Q. Li, Liu, Zhang, & Liu, 2014)
	Synthesis of carotenoids and regulation of the carotenoid biosynthesis pathway in response to high light stress in the unicellular microalga Chlamydomonas reinhardtii	(Couso et al., 2012)
	Functional annotation and sequence-structure characterization of a hypothetical protein putatively involved in carotenoid biosynthesis in microalgae	(Narang et al., 2021)
	Methyl jasmonate-or gibberellins A3-induced astaxanthin accumulation is associated with up-regulation of transcription of β-carotene ketolase genes (bkts) in microalga Haematococcus pluvialis	(Lu et al., 2010)
	Nutrient limitation is the main regulatory factor for carotenoid accumulation and for Psy and Pds steady state transcript levels in Dunaliella salina (Chlorophyta) exposed to high light and salt stress	(Coesel et al., 2008)
	Co-regulation of a gene homologous to early light-induced genes in higher plants and beta-carotene biosynthesis in the alga Dunaliella bardawil	(Lers, Levy, & Zamir, 1991)
	Changes in lipid and carotenoid metabolism in *Chlamydomonas reinhardtii* during induction of CO2-concentrating mechanism: Cellular response to low CO2 stress	(Abreu et al., 2020)
	Time-resolved carotenoid profiling and transcriptomic analysis reveal mechanism of carotenogenesis for astaxanthin synthesis in the oleaginous green alga Chromochloris zofingiensis	(Zhang, Shi, Mao, Kou, & Liu, 2019)
	Transcriptome analysis of carotenoid biosynthesis in Dunaliella salina under red and blue light	(Y. Li, Cai, Gu, & Wang, 2020)
	Transcriptomic analysis of Haematococcus lacustris during astaxanthin accumulation under high irradiance and nutrient starvation	(Kim et al., 2011)
Proteomics	Proteomic analysis of molecular response to oxidative stress by the green alga Haematococcus pluvialis	(Wang, Chen, Sommerfeld, & Hu, 2004)
	Proteome Analysis of Cytoplasmatic and Plastidic β-Carotene Lipid Droplets in Dunaliella bardawil	(Davidi, Levin, Ben-Dor, & Pick, 2015)
	Quantitative proteomic analysis of thylakoid from two microalgae (Haematococcus pluvialis and Dunaliella salina) reveals two different high light-responsive strategies	(Gu et al., 2014)
	Quantitative Proteomics of Chromochloris zofingiensis Reveals the Key Proteins Involved in Cell Growth and Bioactive Compound Biosynthesis	(Qiu, Chen, Wang, Liu, & Lv, 2022)
	Quantitative proteomic analysis of thylakoid from two microalgae (Haematococcus pluvialis and Dunaliella salina) reveals two different high light-responsive strategies	(Gu et al., 2014)
	Proteomic Analysis of the Eyespot of Chlamydomonas reinhardtii Provides Novel Insights into Its Components and Tactic Movements	(Schmidt et al., 2006)
	Proteomic Analysis of the Chlorophyta Dunaliella New Strain AL-1 Revealed Global Changes of Metabolism during High Carotenoid Production	(Ben Amor et al., 2017)
Integrative	Chemical mutagenesis and fluorescence-based high-throughput screening for enhanced accumulation of carotenoids in a model marine diatom Phaeodactylum tricornutum	(Yi et al., 2018)
	Genome and Transcriptome Sequencing of the Astaxanthin-Producing Green Microalga, *Haematococcus pluvialis*	(Luo et al., 2019)
	Novel insights into salinity-induced lipogenesis and carotenogenesis in the oleaginous astaxanthin-producing alga Chromochloris zofingiensis: a multi-omics study	(Mao, Zhang, Wang, & Liu, 2020)
	Development of a stable semi-continuous lipid production system of an oleaginous Chlamydomonas sp. mutant using multi-omics profiling	(Oyama et al., 2022)

Microalgae are a broad microorganism category including nearly 2×10^6^ species that comprise unicellular eukaryotic photosynthetic and prokaryotic cyanobacteria [8]. Photosynthetic microalgae consume atmospheric CO_2_ and light energy to produce a wide range of biomolecules such as proteins, carbohydrates, lipids, microelements, and pigments [9].

Thanks to morpho-physiological and genetic diversity, microalgae have the great potential to produce different metabolites; however, the underlying mechanism is far from being entirely understood which limits its exploitation in strain enhancement approaches using gene editing and genetic engineering. Moreover, the development of new data interpretation, modeling, and validation methods upon the increase of biological data provides a new avenue for addressing these challenges.

Carotenoid production by microalga requires an increase in biomass and rewiring of the metabolic pathways to target metabolites [10]. In the microalgae, systems biology approaches might facilitate the enhancement of β-carotene production. In *Dunaliella salina*, a microalga known for its high β-carotene content, systems biology has played a crucial role in enhancing production. Researchers utilized transcriptomic and proteomic analyses to identify critical enzymes and regulatory genes involved in the β-carotene biosynthesis pathway. By the overexpression of phytoene synthase and the suppression of the lycopene β-cyclase gene, a substantial increase has been achieved in β-carotene yield [11]. Through the integration of multi-omics data, key regulatory mechanisms and metabolic intermediates which control astaxanthin biosynthesis have been identified. Metabolic flux analysis combined with gene expression profiling allowed for the optimization of culture conditions and genetic modifications, leading to significantly increased astaxanthin production [12]. Additionally, in the diatom *Phaeodactylum tricornutum*, systems biology has been employed to enhance fucoxanthin production. By integrating transcriptomic and metabolomic data, researchers constructed a comprehensive model of the fucoxanthin biosynthetic pathway. Targeted genetic engineering based on this model, including the overexpression of fucoxanthin biosynthetic genes and the knockdown of competitive pathways, resulted in elevated fucoxanthin levels [13].

Herein, we focus on recent advances in metabolic pathway reconstruction and integrative analysis to elucidate the underlying networks. This may subsequently help improve carotenoid production by microalgal systems.

## 2 Genomics

Development of the state-of-the-art genomic technologies has pushed the idea of algal application as the cell factories of the desired product. Genomics analysis decodes genome content, architecture, and biosynthetic pathways and provides a blueprint for improving the productivity of algae [9,10]. Publication of the first complete genomic sequence of the model alga, *Chlamydomonas reinhardtii*, facilitates the detection of biosynthetic gene clusters, genomic elements, and functional annotation of genes and enzymes involved in carotenoid biosynthesis in the algae arena [14]. Nevertheless, a large and growing body of literature has investigated and published the genome sequencing and carotenoid pathways characteristics in other microalgae.

**Fig. 1:**
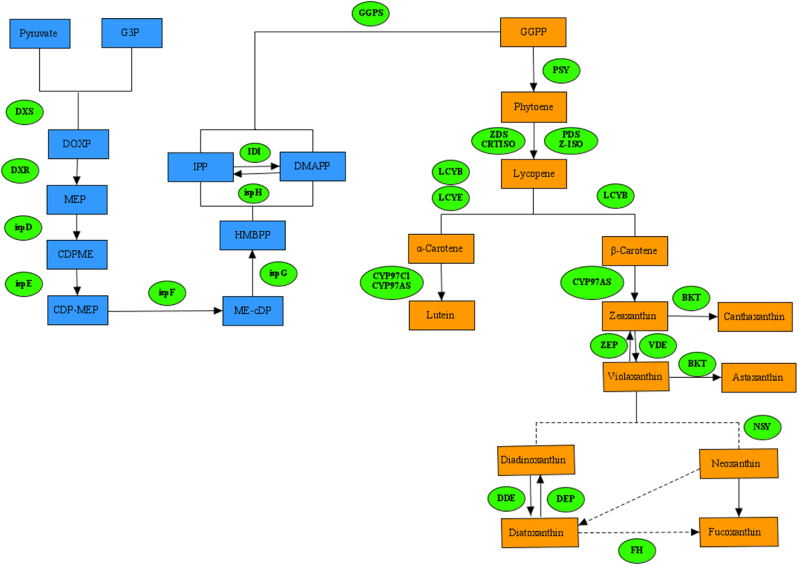
Schematic overview of methylerythritol 4-phosphate/1-deoxyd- xylulose 5-phosphate (MEP/DOXP) and Carotenoids biosynthesis pathway in microalgae.

It was previously reported that enzymes involved in carotenoid biosynthesis were clustered into four major enzyme classes including Transferases, Oxidoreductases, Isomerases, and Lyases [14]. Transferases included the DXS (1-deoxy-d-xylulose-5-phosphate synthase), ispD (2-C-methyl-d-erythritol 4-phosphate cytidylyltransferase), ispE (4-diphosphocytidyl-2-Cmethyl-d-erythritol kinase), GGPS (Geranylgeranyl pyrophosphate synthase), and PSY (15-cis-phytoene synthase) (Fig. 1). In the recent past, different researches have been performed to improve carotenoid production by engineering these transferases. It has been demonstrated that the overexpression of the DXS and PSY enzymes has led to an increase up to 1.5 and 2.0- fold in β-carotene production, respectively (Yang & Guo 2014; Cordero et al., 2011). Oxidoreductases including the DXR (1-deoxy-d-xylulose-5-phosphate reductoisomerase), CYP97C1 (Carotenoid epsilon hydroxylase), ZEP (Zeaxanthin epoxidase), ISPH (4-hydroxy-3-methylbut-2-en-1-yl diphosphate reductase), PDS (15-cis-phytoene desaturase), ISPG ((E)-4-hydroxy-3-methylbut-2-enyl-diphosphate synthase), ZDS (Zeta-carotene desaturase), VDE (Violaxanthin de-epoxidase), CYP97A5 (Beta-carotene hydroxylase), and BKT (β-carotene 4-ketolase) compose another important class of enzymes with a key role in carotenogenesis of microalgae [15]. For example, metabolic engineering of the carotenoid biosynthesis pathway by introducing the *Haematococcus pluvialis* BKT gene in *Chlamydomonas reinhardtii* significantly improved ketocarotenoid production [16]. Likewise, the forward genetic approach corroborated that the PDS enzymes are pivotal for astaxanthin overproduction in microalgae [17,18].

Isomerases including LCYB (Lycopene β-cyclase), IDI (Isopentenyl-diphosphate Delta-isomerase), CRTISO (Carotenoid isomerase), Z-ISO (Zeta-carotene isomerase), and LCYE (Lycopene epsilon-cyclase) and lyses including ispF (2-C-methyl-d-erythritol 2, 4-cyclodiphosphate synthase) further contribute carotenogenesis of microalgae (Fig. 1).

Recently, putative carotenogenic genes in *Nannochloropsis oceanica* were identified, and underlying biosynthesis pathways were reconstructed [19]. It has been demonstrated that the LCYB (Lycopene β-cyclase) overexpression promotes the accumulation of the carotenoids particularly β-carotene, suggesting that the LCYB is rate-limiting for carotenoid biosynthesis pathway in *N. oceanica* [19]. Moreover, a genome-wide approach has been applied to identify the Psy regulatory impacts in *Dunaliella* sp., *Scenedesmus acutus*, and *Diospyros kaki* [20]. More recently, comparative genome mining approaches utilizing bioinformatics resources and algal omics data have been used for motifs, intrinsic physicochemical features, subcellular localization, and pathway analysis of candidate enzymes of the carotenoid biosynthesis and the MEP/DOXP pathways, relevant to the synthesis of carotenoids in microalgal species [21]. It was shown that there are a total of 104 transferases, 193 oxidoreductase, 82 isomerase, and 24 homologous sequences coding for isomerase in different microalgae. Protein family domain analysis of transferases showed that these enzymes harbored the conserved domains of 1-deoxyd- xylulose-5-phosphate synthase, 2-C-methyl-d-erythritol 4-phosphate cytidylyl transferase/ispD, GHMP kinases N terminal domain, Polyprenyl synthetase, and Squalene/phytoene synthase, respectively [21].

Fucoxanthin is an oxygenated carotenoid with potential pharmaceutical and nutraceutical value [22]. Microalgae are among the most promising alternatives for fucoxanthin production with specific advantages [23]. Recently the fucoxanthin biosynthesis pathways were reconstructed in *Isochrysis galbana* by a combination of genome sequencing and conserved domain analysis [24]. It has been highlighted that the metabolic processes of the aforementioned metabolites consist of oxidation, isomerization, acetylation, deep oxidation, hydrogenation, and hydroxylation reactions. The fucoxanthin hydroxylase gene (FH, IZ011859) which catalyzes the hydroxylation of hydrophobic substrates, has a key role in neoxanthin or diadinoxanthin conversion to fucoxanthin. This hypothesis has been confirmed by the overexpression and biochemical assays [24]. Moreover, it has been elaborated that the interconversion cycles of zeaxanthin to violaxanthin (VDE, ZEP) and diatoxanthin to diadinoxanthin (DDE, DEP) consist two distinct epoxidase and de-epoxidase with similar catalytic reactions. Based on the literature, the presence of most epoxidase and de-epoxidase gene proteins play a role in the fucoxanthin metabolic synthesis of *I. galbana* [24] (Fig. 1).

Astaxanthin is a high-value red ketocarotenoid that is synthesized by microalgae. It is thought that β-carotene, as a precursor of astaxanthin, is exported from the chloroplast into lipid droplets where converted to the astaxanthin by the introduction of two hydroxyl groups and two keto-groups by 3,3-hydroxylase CRTR and di-iron betaketolase (BKT) enzymes, respectively [25]. In contrast with *H. pluvialis*, in *Chlorella zofingiensis*, the hydroxylation of β-carotene occurs first and astaxanthin is then formed by the ketolation of zeaxanthin [26]. Genome sequencing and annotation of *Chlorella zofingiensis* provide new insight into carotenoid and astaxanthin biosynthetic pathways in this microalga. It was elaborated that there are four putative carotene hydroxylase genes, encoding three cytochrome P450s (two CYP97A and one CYP97C) and one di-iron type hydroxylase (CHYB). In addition, two putative BKT genes in the genome of *Chlorella zofingiensis* have been found. Whilst, the three other di-iron betaketolase enzymes have been detected in *H. pluvialis* [27]. Multi-copy genes in the carotenoid pathway have specialized and complicated carotenoid production in microalgae by fostering flexibility and adaptability of biosynthetic pathways. Further structural analysis revealed that the BKTs contain highly conserved histidine motifs which are essential for iron binding and the formation of ketocarotenoids in microalgae [28].

Altogether, with the advent of high-throughput sequencing technologies, genomics has provided a better intuition into the genetic basis of carotenoid biosynthesis. It results in the characterization of the genes responsible for a given feature and even the discovery of new genes and enzymes involved in the pathway. This knowledge, in turn, would benefit the downstream omics studies as well as the genetic engineering approach in the development of microalga with high levels of specific carotenoids.

## 3 Transcriptomics

The changes in mRNA levels are often correlated with the increase and decrease of the encoded protein levels. Moreover, mRNA sequences harbor valuable information regarding the flexibility of different synthesis pathways at expression, polymorphism, splicing, and regulatory network up to the optimal culture conditions in microalgae [7,29]. Based on these, the transcriptomic approach which studies the complete set of transcripts has been considered as a vital approach to understand the underlying mechanisms of the carotenogenic response in microalgae. A growing body of literature has shown that abiotic stresses such as high light, quality of light, hormones, salinity, temperature, nutrient deficiency, pH, etc., induce the accumulation of carotenoids in certain microalgae. Carotenoid production is a species- or/and strain-dependent capability that is also affected by the type and severity of the applied abiotic stress along with the growth conditions of the microalgae. However, the underlying mechanisms are not well understood until now. Next-generation sequencing technology has accelerated the research progress on microalgae by increasing our knowledge of stress-responsive pathways. In this regard, a considerable amount of studies have focused on the analysis of carotenoid biosynthesis pathways by transcriptome analysis. Surveys such as that conducted by Li et al. (2020) showed that transcripts involved in the carotenoid metabolism were up-regulated under both red and blue light. They reported that the expression of carotenoid-related genes especially LCY were similarly up-regulated under blue and red light leading to the accumulation of β-carotene in *Dunaliella salina* [30].

Cross-talk of carotenogenesis with phytohormone signalling was dissected using transcriptome analysis [31]. It was shown that the transcript levels of rate-limiting enzymes of carotenoid biosynthesis such as DXS, DXR, IPI, and GGPS were upregulated upon auxin treatment in *Chlorella* sp. BR2 [31]. Previously, similar results had been observed upon ABA treatment [32]. IAA down-regulates the transcripts that are downregulated by ABA [33]; highlighting the fact that ABA signalling is the upstream effector of auxin signalling which interplay with carotenoid synthesis [31]. Nevertheless, differential regulation of PSY genes under IAA and ABA treatment has been documented [31]. Overall, the transcriptome analysis of hormone-treated microalgae corroborates the hypothesis that auxin and ABA induce a basal ABA response, and the production of carotenoids exerted as a fine-tuning mechanism by auxin [31].

The impact of salinity stress on the enhancement of carotenoid production by microalgae has been proven by a considerable amount of literature. For example, the improved accumulation of astaxanthin has been reported under salt stress conditions by *H. pluvialis* and *C. zofingiensis* [34]. Moreover, possible mechanisms of switching the carbon flux to carotenoids in halotolerant microalgae *Dunaliella salina* have been examined using transcriptome analysis under salt stress conditions [7,10].

Up-regulation of the transcripts related to IPI-1, IPI-2, PSY, LycB, crtR-B, BKT2, and crtO (β-carotene oxygenase) under salinity stress in *H. pluvialis* has been documented by Gao et al., 2015. Liang and Jiang (2017) provided a deep insight into the regulatory mechanisms of carotenogenic-related genes under salinity stress by transcriptome analysis [35]. It has been reported that some carotenoid biosynthesis genes such as PSY, LycB, GGPS, CRTISO, and ChyB were up-regulated upon salinity stress treatment in *Dunaliella bardawil*. Deep dissection in *cis*-acting elements of these genes has shown that most of stress-inducible genes contain the salt-regulated elements (SREs), dehydration responsive elements (DREs), cold responsive elements (CREs), hypoosmolarity responsive element (HREs), and light-regulated elements (LREs) in their promoter regions [35]. LREs and W boxes (targeted by WRKY family of transcription factors) were common in all of the above-mentioned genes, whereas, promoters of PSY, LycB, GGPS, CRTISO and ChyB genes contained SREs related motif such as GT1GMSCAM4. It is whilst PDS does not harbour any SREs in its promoter region. Moreover, DRECRTCOREAT motifs which are involved in cold, salt, and drought responsive pathways, were found in the GGPS promoter. More recently, transcriptome analysis on mangrove-isolated *Chlorella vulgaris* UMT-M1 under salinity condition were performed and cross-talk of photosynthesis and carotenoid biosynthesis were highlighted [35].

Alternative splicing during gene expression is a cellular process for transcriptome plasticity in different circumstances [36]. The availability of transcriptomic data and genomic sequences of microalgae offer new possibilities for the analysis of alternative splicing in carotenogenesis-related genes and patterns of expression at the exon level as another layer of gene regulation. In this regard, we surveyed the alternative splicing pattern of *Auxenochlorella protothecoides* during the transition from autotrophic to heterotrophic growth conditions. Results indicated that the heterotrophic condition affects alternative splicing patterns of carotenoid biosynthesis genes. It has been shown that the PDS, LCYB1, CrtISO, ZEP, P450, CHY, ZDS, and LCYE genes differentially undergo alternative splicing processes at two autotrophic and heterotrophic conditions with different carotenoid accumulation rates. Turning to the evidence of our results [29] indicates that exon usage as a regulatory mechanism is an important factor in carotenoid accumulation conditions.

Transcriptomics provides appropriate information on gene expression levels, patterns, and regulation under different growth and suboptimal conditions. It enables the identification of regulatory mechanisms controlling the pathway and the development of metabolic engineering strategies for carotenoid biosynthesis manipulation. By overexpressing or silencing specific genes of the pathway, researchers can increase the production of desired carotenoids or redirect the flux toward the synthesis of novel carotenoids.

## 4 Proteomics

**Fig. 2:**
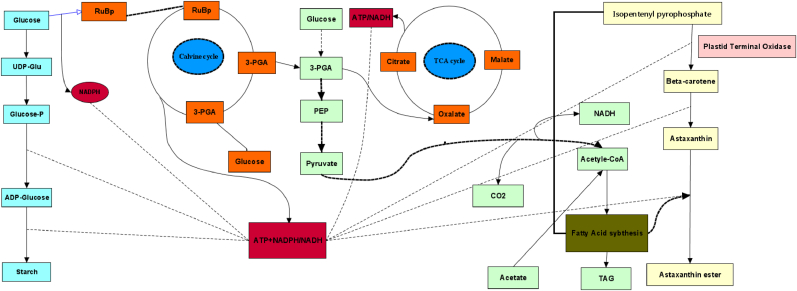
Schematic representation of light responsive pathways related to astaxanthin biosynthesis in *H. pluvialis*. Dotted line represent the energy carrier production and involvement in the pathway, the thin line represent the metabolic pathways involved in beta-carotene production and thick arrows highlighted the main route for the biosynthesis of carotenoids in microalgae.

The proteomic approach complements the insufficiency raised from the individual use of genomics and transcriptomics in understanding the complex biology of microalgae. Proteomics discloses protein-protein interaction, post-translational modification, and subcellular localization. By providing this information on proteins, proteomics delves into the mechanisms of biological processes and network functions [37]. The first proteomic analysis in microalgae was conducted in the unicellular freshwater green alga C. reinhardtii, chosen as a model organism. Proteomic studies based on two-dimensional electrophoresis (2-DE) and Liquid Chromatography with tandem mass spectrometry (LC-MS) have been reported as the main techniques of separation and characterization of protein structure which decipher the underlying mechanisms of carotenoid accumulation in microalgae. This approach successfully has been applied to depict the photosynthetic acclimation of *H. pluvialis* and *D. salina* and carbon flow redirection to carotenoid biosynthesis under high light stress (Fig. 2) [38].

Recent proteome analysis of microalgae revealed some hidden layers of light stress-responsive mechanisms in microalgae [38]. Under the high intensity of light, due to a decrease in the activity of cytochrome *b*6/f complexes, photosynthetic electron transport (PET) pathways were destroyed. It led to diminishing reduction potential of photosynthesis machinery, as a force of the Calvin cycle triggering. Then, the regeneration of Ribulose 1,5-bisphosphate was suspended to break the reduction power constraint of the Calvin cycle for carbon fixation. Afterwards, complementary pathways including starch, and subsequent astaxanthin and fatty acid biosynthesis pathways were triggered (Fig. 2) [38]. Moreover, pentose phosphate and glycolysis were up-regulated [39]. In line with the above-mentioned mechanism, the correlation of reduced Rubisco activity with astaxanthin synthesis has been proven by Chen et al. (2022) in oxidative stress conditions [24].

More recently, LC-MS/MS-based tandem mass tag (TMT) approach was applied to reveal the key proteins involved in carotenoid biosynthesis in *Ch. zofingiensis* microalgae [40]. It has been shown that glucose supplementation induces biomass and astaxanthin accumulation in *Ch. zofingiensis* and *Chlorella vulgaris* [41]. Regarding the underlying mechanisms of carotenoid accumulation upon glucose supplementation, it was demonstrated that the glucose modulates the transcription of the BKT and CHYb through de novo protein synthesis [42]. In accordance to these findings, it has been reported that fatty acid accumulation diminishes glucose supplementation [40].

In another study, two-dimensional electrophoresis was integrated with MS/MS spectrometry analysis and peptide fingerprinting to interpret the molecular mechanism behind carotenoid production in *Dunaliella* sp. AL-1 [43]. Their results showed that the rate of CO2 assimilation by photosynthetic was reduced in the carotenoids accumulation circumstance; however, the protein level of carbonic anhydrase, which catalyzes the first step of CO2 fixation in the photorespiration process, was up-regulated under nitrogen deficiency, salinity, and high light intensity conditions. Another important finding was that the ribulose phosphate-3-epimerase, a key enzyme of the Calvin cycle, was down-regulated, leading to the accumulation of xylulose-5P and triggering the pentose phosphate cycle under the carotenoid accumulation condition. Moreover, it was demonstrated that the upregulation of enolase, at the above-mentioned condition, stimulates the accumulation of the 2-phosphoglycerate to phosphoenolpyruvate and pyruvate, the end product of glycolysis.

Overall, analysis of microalgal proteome under different conditions enables the researchers to identify the involved proteins in carotenogenesis and understand protein-protein interactions, networks, and their regulation. Proteomics has also allowed for the identification of post-translational modifications of regulatory importance. Through the acquired knowledge it would be possible to develop strategies for optimization of microalgal carotenoid production for various industrial applications.

## 5 Integrative approach

**Fig. 3:**
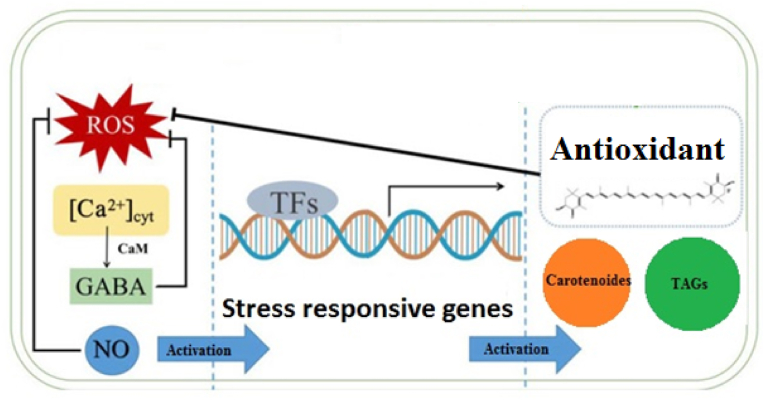
Cross talk between Ca2+ signaling pathway, ROS and Carotenoid biosynthesis pathways under stress condition.

Given the extensive application of high-throughput sequencing of microalgae in metabolite accumulation conditions, the amount of deposited omics data is significantly increasing. A key characteristic of system biology and high-dimensional biological data is the ability to integrate multiple datasets to generalize the regulatory mechanism and come up with precise biological conclusions. An integrated meta-analysis can overcome individual variation challenges, and improve mild data perturbations by combining related hypotheses [[44], [45], [46]]. In microalgae, this implies a more powerful approach to discovering the condition-specific co-regulations and functions than regulations of individual experiments [7]. Moreover, this approach provides valuable knowledge regarding the functional modules related to conditional specific responses within and between the species. In this regard, we integrated cross-species RNA-sequence data of meta-analysis with supervised machine-learning models to elucidate and prioritize the stress-responsive pathways in microalga *Dunaliella* [7]. Results of our study indicated that the structural proteins of the photosynthesis apparatus, chaperone-mediated autophagy, and ROS-related genes are the central backbones of *Dunaliella* salt stress-responsive pathways. Additionally, the cross-talk between Ca2+ signal transduction and ROS signalling network with carotenoid accumulation was proposed (Fig. 3) [7].

**Fig. 4:**
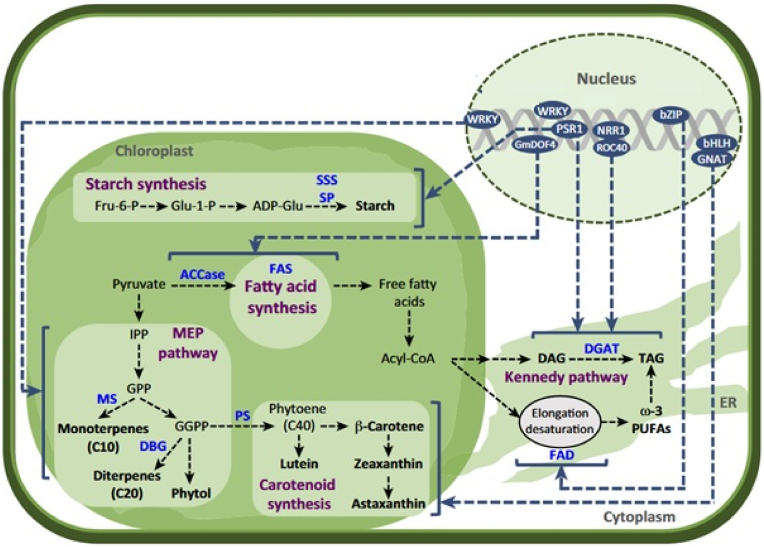
Activation of carotenoids biosynthesis pathways by transcription factors (TFs) under stress condition. Dotted line represents the interaction of different elements in the regulatory network predicted in microalgae (Bajhaiya et al., 2017).

The majority of microalgae transcriptome studies of carotenoid accumulation have solely focused on the detection of the differentially expressed genes in different conditions. Whilst, they ignored the degree of interconnection between different genes and/or their relevant protein product. This is while the genes with similar expression patterns may be functionally important [44,45,47]. Harnessing the network-based approaches to discover the transcriptional circuits and master regulators of specific metabolic processes in different stress conditions has been reported [48]. Meanwhile, hub genes in these constructed networks represent the essential genes related to specific phenotypes [7,44]. Co-expression network analysis which is based on the guilt-by-association paradigm defines two genes with correlated expression patterns across stress and developmental conditions [49]. These approaches were successfully applied to identify the more important hub/essential genes and functional important modules related to carotenoid biosynthesis in *Auxenochlorella protothecoides* and *Dunaliella salina* [10,48]. It was reported that the expression pattern and connectivity characteristics of carotenoid biosynthesis genes were changed during the transition of microalgae from autotrophic to heterotrophic growth conditions. Functional annotation of non-preserved modules of constructed modules also provided new insight into the interaction reforming of carotenoid biosynthesis genes with some specific transcription factors such as SBP, C3H and MYB_related, GARP-G2-linke and protein kinases (PKs) such as GNAT (GCN5-related-N-acetyltransferase), SNF2 (sucrose non-fermenting 2) (Fig. 4) [48].

Based on what was mentioned, the integrative omics approach consisting of a comprehensive analysis of the molecular mechanisms uncovers the gene functions, regulatory mechanisms and interconnection of metabolites and enzymes with carotenoid biosynthesis pathways in microalgae. A study using an integrative omics approach in the green microalga *Dunaliella salina* revealed that the expression of genes involved in carotenoid biosynthesis is regulated by a complex network of transcription factors and epigenetic modifications [50]. The integrative omics approach in the microalgae revealed the interconnection of different biological pathways with the carotenoid biosynthesis pathway.

In conclusion, the integrative omics approach has greatly advanced our understanding of the carotenoid biosynthesis pathway, its regulation, and its interaction with other metabolic pathways in the microalgae. This knowledge can be used to develop novel strategies for the optimization of carotenoid production in microalgae for various industrial applications.

## 6 Conclusion

Our review summarizes and discusses the recent progress and knowledge regarding the carotenoids biosynthesis pathways in microalgae. Integrative systems biology-driven insights regarding the regulatory mechanism of carotenoid accumulation in response to different stress conditions were reviewed. Moreover, cross-talk of carotenogenesis with different biological processes such as phytohormone signalling was discussed. The employment of these approaches proposed some co-regulatory modules and hub genes related to different carotenoids such as beta carotene, astaxanthin, and fucoxanthin. The current study provides comprehensive insights into the underlying mechanisms of carotenogenesis and valuable information for the optimization of different strategies of carotenoid accumulation in future efforts.

## Data availability statement

No data was used for the research described in the article.

## CRediT authorship contribution statement

**Bahman Panahi:** Writing – original draft, Visualization, Data curation, Conceptualization. **Nahid Hosseinzadeh Gharajeh:** Writing – review & editing, Writing – original draft. **Hossein Mohammadzadeh Jalaly:** Writing – original draft, Visualization. **Mohammad Amin Hejazi:** Writing – original draft.

## Declaration of competing interest

The authors declare that there is not any conflict of interest.
